# Bisphosphonate therapy in pediatric patients

**DOI:** 10.1186/s40200-014-0109-y

**Published:** 2014-12-17

**Authors:** Guiti Eghbali-Fatourechi

**Affiliations:** Affiliate Professor of Endocrinology and Metabolism Research Institute, Tehran University of Medical Sciences, Tehran, Iran; Affiliate Faculty of University College of Omran and Tosseh, Hamedan, Iran

**Keywords:** Bisphosphonate, Nitrogen-containing bisphosphonate, Pediatric osteoporosis, Pediatric bone loss, Osteogenesis imperfecta, Corticoid-induced osteoporosis, Pediatric bone malignancy

## Abstract

Although for many decades bisphosphonates were used for adult bone loss, bisphosphonate administration in pediatric patients is new and was initiated in the past 15-year. The indications for pediatric bisphosphonates was extended to childhood malignancies with bone involvement, after additional effects were unveiled for bisphosphonates with recent research. In this article we review childhood bone loss and conditions with bone involvement in which bisphosphonate therapy have been used. We also review mechanisms of action of bisphosphonates, and present indications of bisphosphonate therapy in pediatric patients based on results of clinical trials.

## Introduction

The use of bisphosphonate therapy in pediatric patients was suggested in 1998 when the cyclic administration of intravenous pamidronate in children with osteogenesis imperfecta resulted in reduction in bone resorption, increase in bone density, and reduction in fracture incidence [1]. Since the mechanism of action in children is different from adults, as bone in children is a growing tissue and responds to bisphosphonates differently than the adult bone, it is difficult to extrapolate the adult bisphosphonate therapy regimens to the pediatric patients. Two years after discontinuation of pamidronate therapy, older teens with osteogenesis imperfecta maintained their spine bone mass, while the bone mass declined in younger teens. The persistence of gains in BMD after bisphosphonate therapy appeared to be dependent on the age of the children and the amount of regional residual bone growth [2]. In 2007 and 2014 a Cochrane Database Systemic Review [3–5] concluded there were insufficient data to support the use of bisphosphonates as standard therapy in children. Importantly, although many clinical trials in pediatric patients reported significant gains in BMD and decrease in pain compared to placebo with bisphosphonates, they did not conclude that bisphosphonates significantly reduce the incidence of fractures in osteogenesis imperfecta [4], neither bisphosphonates improve survival of cancer patients. However, safety and efficacy of short-term therapy (≤2-yr) is sufficient to justify their use in severe cases of bone loss such as osteogenesis imperfecta and pediatric bone malignancies associated with fracture and pain.

## Molecular mechanisms of actions of bisphosphonates

The molecular mechanisms of action of bisphosphonates were described recently. All bisphosphonates are characterized by 2 phosphonate groups sharing a common carbon atom (P-C-P) backbone, responsible for the strong affinity of bisphosphonates for bone mineral, the hydroxyapatite. The adsorption of bisphosphonate molecule to bone mineral is responsible for the uptake and retention of bisphosphonate on the skeleton, its diffusion and storage within the bone, and its potential release from bone [6].

*The simple bisphosphonates* attached to calcium are taken up by osteoclasts by endocytosis and are incorporated as toxic, non-hydrolysable metabolites, methylene-containing ATP analogues. Methylene-containing metabolites, or ATP analogues, contain the P-C-P groups of bisphosphonates in place of the pyrophosphate (P-O-P) moiety of ATP. ATP analogs are resistant to hydrolytic breakdown and the release of phosphate [7]. Metabolites of simple bisphosphonates closely resemble proton pump inhibitors (PPi), and as such, can be incorporated into the active site of aminoacyl-tRNA synthetase enzyme in the cell. These cytotoxic metabolites condensate and accumulate in the cytosol of osteoclasts and cause apoptosis of these cells. No other cell type can acidify the bone surface, a condition required for this adsorption [8–10]. It was recently found that monocytes and macrophages also were able to internalize bisphosphonates, but only transiently. On the contrary, RANKL and TNF alpha can prevent the bisphosphonates apoptosis and restore osteoclast’s bone resorption activities [9,10]. In summary, simple bisphosphonates act as pro-drugs, absorbed by osteoclasts where they accumulate as toxic metabolites and cause apoptosis of osteoclasts and prevent the bone resorption.

*Nitrogen-containing bisphosphonates* (N-bisphosphonates) are up to several magnitude more potent than simple bisphosphonates and they inhibit osteoclasts using a different pathway [11]. N-bisphosphonates inhibit enzymes of cholesterol synthesis, the mevalonate enzyme pathway and the farnesyl diphosphate synthase, within osteoclasts. The inhibition of these enzymes prevents the prenylation of small GTPases and causes unprenylated GTPases. Accumulation of unprenylated GTPases modifies important functions in osteoclasts including membrane trafficking and ruffling, and induces apoptosis of these cells [12]. Zoledronic acid is the most potent inhibitor of farnesyl diphosphate synthase and has the highest affinity for hydroxyapatite and the longest duration of action [13]. The inhibition of bone resorption by N-bisphosphonates is not associated with signs of cell toxicity or decrease in OC numbers at therapeutic doses. Instead, N-bisphosphonates can lead to the formation of “giant” hyper-nucleated OC associated with resorption lacunae, seen as functionally inactive pre-apoptotic osteoclasts [14,15]. Bisphosphonates indirectly oppose key mediators of osteoclast function and survival, RANK/ RANKL, by increasing osteoprotegerin (OPG) production. Increase in OPG opposes the binding of RANKL to the RANK receptor [16–19]. In addition to anti-osteoclastic effects, bisphosphonates have antitumor properties. In pre-clinical trials in neuroblastoma, it is shown that zoledronic acid stimulates tumor-specific T cells by enhancing the anti-tumor activity of natural-killer cells [20]. In clinical trials, zoledronic acid combined with conventional chemotherapy, decreases the production of IL6, which is associated with poor-outcome of neuroblastomas [21]. Despite a decrease in bone remodeling, bone formation parameters are maintained because osteoblasts remain active, resulting in a positive remodeling balance [22].

### Effects of bisphosphonates on pediatric patients

In children with osteogenesis imperfecta, the most studied cause of pediatric bone loss, intravenous pamidronate therapy increases the size of vertebral bones and reshapes pre-existing vertebral compression fractures. Older children with lower bone density gains more in BMD than younger, although younger children have less deficit in BMD at base [23]. Two years after discontinuation of intravenous pamidronate, areal BMC Z-scores in osteogenesis imperfecta children remains above pretreatment levels but below normal levels [24]. Trans-iliac histophotometry after 2 years intravenous pamidronate therapy shows maximal increases in cortical and cancellous bone thicknesses, with considerable increases in trabecular number [25]. The cortical width of iliac bone almost doubles during the first 2 years of pamidronate therapy, but changes little when therapy is continued for another 2-year. These results suggest stored bisphosphonates maintain their biological activity at least 2 years after discontinuation.

Although in adults, bisphosphonates appear to suppress bone resorption up to 10 years after discontinuation, in younger children because of higher bone turnover, recovery of recycled bisphosphonates from bone is shorter. Children with osteogenesis imperfecta treated with bisphosphonates at early age have normal or improved growth and new bone acquisition is reported in studies on long bone fractures after discontinuation of pamidronate therapy. Bisphosphonates at usual therapeutic doses do not permanently apparently disable the bone turnover mechanisms, neither usual therapeutic doses result in osteopetrosis. Overall, pamidronate therapy decreases bone remodeling, however bone formation parameters are less inhibited than bone resorption parameters, resulting in a positive remodeling balance [22]. Urinary NTX excretion decreases significantly with bisphosphonates and increases slightly after discontinuation above normal healthy levels, but it remains well below pretreatment levels [23]. Neither radiological changes in bone density nor urinary NTX are correlated with changes in BMD [25].

The improvement of BMD with only one infusion of zoledronic acid every 6 months for 1 year, and 3 monthly infusion for 12 months, were similar in pediatric spinal cord injury [26]. Also, one single infusion of zoledronic acid in adult patients reduces >30% the rate of clinical fractures over 3 years of follow up, compared with placebo [27]. These important observations, points to the possibility of shorter safer length of therapy, longer dose interval with sustained effects, and a better acceptance by patients.

The safe upper limit dose for each entity of bisphosphonate is not yet established [3]. A 2014 Cochrane Systemic Review assessed the effectiveness and safety of bisphosphonate therapy. Effect on fracture reduction was inconsistent and was observed in only two trials with statistically significant difference between oral or intravenous bisphosphonates compared to placebo control. All trials reported statistically significant increase in lumbar spine BMD Z-scores. The outcome between zoledronic acid and intravenous pamidronate were not different. Neither oral nor intravenous bisphosphonates improved significantly bone pain, growth, and functional activity versus placebo. Because of the limited number of controlled clinical trials and small number of pediatric patients the Systemic Review did not confirm that bisphosphonates decrease consistently fracture rate, nor they consistently improve pain or the functional mobility [5].

### Adverse effects of bisphosphonates in pediatric patients

Bisphosphonates are generally well tolerated in pediatric patients. Adverse effects are limited, and are predictable based on previous trials. In most cases “acute phase reaction” is observed with fever, malaise, abdominal pain, vomiting, muscle or bone pain with the initiation of either intravenous or oral agents within 1–3 days, and lasting few days [1,28]. Asymptomatic hypophosphatemia, and hypomagnesaemia and hypocalcemia causing tetany are rare and prevented with supplementation with calcium and vitamin D [29].

More serious side effects seen in adults including uveitis, thrombocytopenia, esophageal or oral ulcerations, are rare in children. One case of uveitis was reported among 19 children with Langerhans cell histiocytosis treated with bisphosphonates in a retrospective study in Japan [30]. Avascular necrosis of the jaw seen in adult [31] is not seen in pediatric patients. Severe case of respiratory distress syndrome was reported with initiation of pamidronate in an infant with history of airway disorders [32]. Osteomalacia was seen in an adolescent with fibrous dysplasia after intravenous cyclic pamidronate therapy [22].

Long term retention of high-affinity bisphosphonates is the major concern in young girls [33–35]. In experimental studies, bisphosphonates readily crossed the placenta. Thus bisphosphonates can possibly affect fetuses and cause hypocalcemia. Skeletal anomalies in offspring is seen in animal models [36]. A pregnancy test is recommended before therapy in teenage young women. However, full extent of fetal risks is still unknown in humans because of small number of fetuses exposed. In two infants delivered to mothers treated with bisphosphonates, asymptomatic hypocalcemia without any skeletal anomaly was reported in the newborn [35].

The greatest concern in the young patients is long term suppression of bone turnover. Osteopetrosis and pathological fractures were developed in a 12 year child treated for idiopathic hyperphosphatemia, treated with high doses of pamidronate for 33 months (up to 100 mg intravenously every three weeks) [37]. Transient and dose-dependent inhibitory effects on bone length and growth were also observed in mice after zoledronic acid therapy [38]. In a phase I trial of zoledronic acid combined with cyclophosphamide in neuroblastomas, two cases of osteosclerosis out of 21 children were reported [21].

### Pediatric bone disorders: candidates for bisphosphonate therapy

The major indication for bisphosphonates in pediatric patients is osteoporosis either primary or secondary. Additional antitumor properties of bisphosphonates have recently added a second indication for bisphosphonates as adjuvant medication with chemotherapy in pediatric bone malignances.

*Definition of childhood osteoporosis:* Fractures with minor trauma in apparently healthy children might be a complication of unrecognized disorders with bone loss [39]. One out of 3 otherwise healthy children fractures by age 17 [40–42]. The 2013 revised position of International Society for Clinical Densitometry (ISCD) defines osteoporosis in children by two criteria 1) the presence of a significant fracture history indicated by either one or more vertebral compression (crush) fractures in the absence of local disease or trauma or, two or more long-bone fractures by age 10 or, three or more long-bone fractures at any age up to age 19; and 2) a low bone mineral content and areal bone mineral density (aBMD) with BMC/BMD Z-score ≤ −2.0 SD [43] [http://www.iscd.org] 2013 Pediatric Official Positions]. However aBMD Z-score ≥ −2.0 SD does not exclude osteoporosis in high risk patients since long bone or vertebral compression fractures might occur with low impact trauma. The posterior-anterior (PA) spine densitometry and a “total body less head” (TBLH) are the preferred skeletal sites for BMC and aBMD measurements. ISCD has recommended that the time interval between 2 DXA be not less than 6 months.

*Primary pediatric osteoporosis* result from intrinsic skeleton abnormalities, such as heritable disorders. The most common cause of primary pediatric osteoporosis is osteogenesis imperfecta, an inherited disorder characterized with bone fragility and low bone mass with an incidence of 1/10,000 births, caused by mutations of two genes that encode collagen type I alpha chain, *COL1A1* and *COL1A2* genes [44]*.* There is a wide variety of clinical severity and phonotypes, associated with a wider variety in genetic characteristics [45]. The four initial clinical types of osteogenesis imperfecta as described by Sillence [46,47] are type I with no bone deformity, type II lethal in perinatal period, type III the most severe form in children surviving the neonatal period, with extremely short stature and short limbs, multiple spinal fractures and secondary deformities, and type IV with less severe bone deformities and variable short stature. Three additional types were later described with the same phenotypes as the first four types but with mutations not related to collagen genes and are types V, VI, and VII [48–51].

Bruck Syndrome and Ehlers-Danlos syndrome are rare heterogeneous autosomal recessive disorders phenotypically similar to osteogenesis imperfecta characterized by bone fragility. The genetic mechanism however consists of posttranslational modifications of collagen *COL3A1* gene that result in an aberrant cross-linking of bone collagen due to under-hydroxylation [52–55].

Marfan syndrome, an autosomal dominant heritable disorder, results from mutations in the *FBN1* gene, which encodes fibrillin-1, an extracellular matrix component found in micro-fibril structures characterized by wide variety of skeletal, ocular, and cardiovascular anomalies [56,57]. Osteoporosis-pseudoglioma syndrome is a recessive autosomal genetic disorder involved with primary severe childhood osteoporosis and visual disturbances. Mutations in the low-density lipoprotein receptor-related protein 5 gene (LRP5) have been frequently detected [58].

*Secondary childhood osteoporosis* result from divers processes outside of the skeleton. They comprise neuromuscular disorders associated with immobilization such as cerebral palsy, Duchenne muscular dystrophy (DMD), and spinal cord injury. DMD is an X-linked disorder arising from mutations in the dystrophin gene, which encodes a structural muscle fiber protein. In DMD association of severe muscle weakness, impaired motility, might result in high incidence of vertebral compression fractures sometime aggravated with corticoid therapy [59,60].

Chronic illnesses such as eating disorders, liver failure, and coeliac disease, chronic inflammatory conditions including inflammatory bowel disease, systemic lupus erythematosus, HIV, renal failure, and severe burns also cause bone loss.

Secondary amenorrhea are also associated with secondary osteoporosis and include functional hypothalamic amenorrhea characterized by the dysfunction of the hypothalamic-pituitary-ovarian axis, absence of functional or anatomical lesion, associated often with stress, weight loss, or excessive exercise [61,62]. In this syndrome, suppression of gonadotropin-releasing factor (GnRF) pulsatility, over-activity of hypothalamic-pituitary-adrenal axis, increased secretion of corticotropin-releasing hormone, disturbance of the hypothalamic-pituitary-thyroid axis [63], and estrogen deficiency [62] are present. The “female athlete triad” refers to amenorrhea, osteoporosis, and poor nutritional behavior, seen in exercise-induced amenorrhea [64,65]. Eating disorders are 10 times more common in females and result in secondary amenorrhea in girls and low testosterone in boys. Osteopenia, osteoporosis, shorter stature, and high rates of stress fracture characterize anorexia nervosa [66,67]. Other endocrine and reproductive disorders with hypogonadism and secondary osteoporosis include Turner syndrome, growth hormone deficiency, hyperthyroidism, diabetes, hyper-prolactinemia, and glucocorticoids excess (Cushing syndrome). Causes of iatrogenic osteoporosis include osteoporosis associated with glucocorticoid [68,69], methotrexate, cyclosporine, radiotherapy, GnRH agonists, T4 suppressive therapy and finally, anticonvulsants therapy. Thalassemia, a hereditary anemia resulting from defects in hemoglobin synthesis, is among the most common genetic disorders with a worldwide incidence of 4.4 per 10,000 birth [70,71]. The incidence of osteopenia or osteoporosis is estimated from 60 to 90% starting in early age, with significant decrease in OPG/RANKL ratio [72] and imbalance between bone formation and bone resorption [73,74]. Inborn errors of metabolism associated with bone loss and osteoporosis include glycogen storage diseases, galactosemia, Gaucher disease, and homocystinuria.

Langerhans cell histiocytosis is a chronic proliferative disease characterized by uncontrolled clonal proliferation of CD1a + dendritic Langerhans cells, mostly proliferating in bone tissue [30].

*Pediatric malignancies* with bone involvement result in significant pain and increased morbidity and mortality [75]. The key mediator of osteoclasts survival, RANKL [16–19] is secreted by both osteoclasts and by malignant cells and promotes the bone resorption [76].

Neuroblastoma, the most frequent extra-cranial tumor in children has a bone metastasis rate of 56% that is generally present at diagnosis, increasing the morbidity and mortality [19]. High peripheral blood level of IL6 is associated with poor-outcome of neuroblastoma, and is believed to stimulate the invasion of osteoblastoma tumor cells to bone microenvironment. IL6 levels decrease with zoledronic acid combined with cyclophosphamide in pediatric patients with neuroblastoma [20,21].

Ewing’s sarcoma, the second most frequent bone malignancy behind osteosarcoma in pediatric and adolescent patients, in 85% of cases, is defined by a chromosomal translocation of t(11;22)(q24;q12) [17]. Osteosarcoma, if localized at diagnostic, and when treated, has a 5-year overall survival rate of 70%, that drops to < 15% in patients with multifocal disease or relapse [17].

## Initial and supportive therapy of pediatric bone disorders

Management of the primary disorders generally improves childhood osteoporosis. Weight control in eating disorders, early management of amenorrhea in anorexia nervosa [67], physical therapy in children with restricted mobility [77], correction of hemoglobin levels by transfusion and prevention of iron overload in thalassemia with iron chelating [71,78], are the initial steps. Adequate daily intake of calcium and vitamin D are necessary. In osteogenesis imperfecta, lower serum 25OH-D levels are associated with both lower LS-aBMD Z-scores and with higher urinary bone turnover indexes (urinary NTX/Cr). Every 1 nmol/liter increase in 25OH-D increases the Z-score by 0.008. The current conservative consensus is to maintain serum 25OH-D levels above 50 nmol/liter (20 ng/ml) [79]. The 25OH-D levels are also inversely associated with PTH concentrations [80]. Recombinant hPTH therapy, the most effective anabolic agent in management of adult osteoporosis, has caused osteosarcomas in growing animal models and its use in children is not acceptable [81].

Genetic regulations of RANK/RANKL [82] and osteoprotegerin (OPG) [83] or stimulation of production of OPG that decreases RANKL/OPG ratio [16–18], has been considered in metastatic bone cancers. The administration of sex steroids in anorexia nervosa does not increase the BMD in adults [84] and it is not recommended in children.

## Selected pediatric conditions treated with bisphosphonates

We have summarized major recent clinical trials, which studied bisphosphonate therapy in pediatric patients, in Table 1. Major pediatric conditions treated with bisphosphonates are discussed below:

**Table 1:** **Selected trials with bisphosphonate and doses used, p value of spine BMD and fracture reduction rates, compared to control**

**Year**	**Author**	**Disease**	**Age**	**Trial type**	**Duration of therapy**	**Bisphosphonate**	**Doses**	**Spine BMD vs CTL**	**Fr Reduction vs CTL**
1998	Glorieux FH	OI	range 4 - 15	Uncontrolled,	5 year	IV PAM	6.8 ± 1 mg/kg/year	p < 0.001	p < 0.001
				Observational					
2000	Plotkin H	OI	< 3	Controlled	1 year	IV PAM	12.5 mg/kg/year	p < 0.001	p < 0.01
2005	Letocha AD	OI	range 4 - 13	Randomized,	1 year	IV PAM	1 mg/kg/d × 3d/3 M	p = 0.054	p = 0.04
				Controlled					
				Observational	2 years				
2005	Golden H	Aneroxia N	range 15 - 19	Randomized	1 year	Oral ALN	10 mg/d	p = 0.02	
				Double-Blind,					
				Placebo-controlled					
2005	Rudge, S	Corticoid-in.	range 4 - 17	Randomized	1 year	Oral ALN	1-2 mg/kg/week	p = 0.013	
		osteoporosis		Double-Blind,					
				Placebo-controlled					
2010	Bachrach SJ	C P	mean 11	Controlled	13 months	IV PAM	unspecified		p = 0.02
				Observational	mean 4 years				
2011	August KJ	Cancer relapse	mean 12.5 y	Retrospective	3 years	IV ZOL	>10 y 4 mg		no Statistics
			range 1–23 y				<10 y 0.08-0.16 mg/kg		
2011	Ward LM	OI	range 4 - 19	Randomized	2 years	Oral ALN	<40 kg 5 mg/day	p < 0.001	p = 0.07
				Double-Blind,			>40 kg 10 mg/day		
				Placebo-controlled					
2011	Russel HV	Neuroblastoma	mean 7.5 y	Observational	capped at 4 mg	IV ZOL	2 mg/m2	Prolonged stability	
		reccurent	range 0.8 - 25	Dose limiting toxicity		Escalating doses/28-d	3 mg/m2	no Statistics	
		NANT				(+ Cyclophos)	3-4 mg/m2		
2013	Bishop N	OI	4 - 15	Randomized	1 year	Oral Risedronate	2.5 mg/d or 5 mg/d	p < 0.0001	p = 0.044
				Multicenter	Observational years 2-3				
				Double-Blind,					
				Placebo-controlled					

*IV PAM* Intravenous pamidronate, *ALN* Alendronate, *IV ZOL* zolendronic acid, *Fr* Fracture, *Neurobl* neuroblastoma, *CP* cerebral pulsy, *conrticoid-in* corticoid-induced osteoporosis, *cyclophos* cyclophosphamide.

### Osteogenesis imperfecta

In an uncontrolled observational study in children with severe osteogenesis imperfecta, intravenous pamidronate 6.8 ± 1.1 mg/kg/year was administered up to 5 years. There was a mean annual increase in BMD of 42 ± 29%, with increase in Z-score increment of about 2 points. There was also improvement of mobility, and the fracture rate decreased compared to the rate before therapy [1].

A clinical trial analyzed bone densitometry of boys and girls aged 2 weeks to 17 with osteogenesis imperfecta types I, III, and IV, who received a 4-year intravenous pamidronate compared to “untreated” age- and type-matched controls. Patients with ages < 2 years received 0.25 mg/kg day 1, and 0.5 mg/kg days 2 and 3 every 8 weeks. Ages 2–3 years received 0.38 and 0.75 mg/kg day 1 and days 2–3 respectively, every 12 weeks. Those ages >3 years received 0.5 and 1 mg/kg/day, day1 and days 2–3 respectively, every 16 weeks. Total yearly doses in the 3 groups were the same. Results concluded that spine aBMD Z-scores, BMC, bone volume, and volumetric BMD significantly increased with intravenous pamidronate compared to control untreated children (p < 0.001 for each). The treatment was associated with both increased cortical thickness and increased trabecular compartment evidenced by both radiological and histophotometric analyses. Results suggested the most severely affected patients at the baseline were the older children and they were the ones who benefited more from intravenous pamidronate therapy [23]. Changes in serum alkaline phosphatase and urinary NTX did not correlate with gains in bone mineral density. The type of collagen mutations had no influence on results of the study [22]. Also transiliac histophotometry indicated that cancellous bone volume and cortical width initially increased significantly but changed little after year 2 of therapy [25]. To compare the effectiveness of intravenous versus oral bisphosphonate therapy, a 2-year oral alendronate therapy was also used in prospective, randomized, double-blind placebo-controlled, multicenter trials in boys and girls aged between 4 and 18 with osteogenesis imperfecta. Oral alendronate was given at 5 mg/d and 10 mg/d doses in children < 40 kg and >40 kg respectively, for 2 years. Oral alendronate significantly increased lumbar spine BMD Z-scores mostly in the first year, and suppressed sharply urinary bone resorption index, the urinary NTX the first 6 months then changed little the remaining time compared to control (p < 0.001 for each). The incidence of long-bone fractures, the average vertebral heights, the cortical thickness, bone pain and finally functional activity were similar to the control placebo group [28]. Taken together the study concluded that the fracture outcome, bone pain, and quality of life were improved with oral alendronate and were comparable to intravenous bisphosphonates at doses used [28].

A controlled trial treated severely affected osteogenesis imperfecta children under 3 years of age for a period of 12 months with intravenous pamidronate in 3 consecutive days for four to eight cycles, with an average cumulative dose of 12.4 mg/kg. The age-matched, severity-matched controls did not receive the treatment. BMD in treated children increased dramatically up to 227% (p < 0.001), and fracture rate decreased significantly (p < 0.01) compared to untreated control children [85]. Side effects were minor and as expected. This trial confirmed the intravenous pamidronate in very young and severely affected osteogenesis imperfecta children is safe and beneficial.

Intravenous pamidronate 10 mg/m2 per day administered for 1 year to types III and IV osteogenesis imperfecta children in a randomized controlled trial, increased significantly lumbar spine BMD Z-scores and volumetric vertebral size. Fracture rates decreased in upper extremities and not in the lower extremities, functional mobility and pain were not improved, and a second year extension of the therapy did not additionally significantly improve the bone density [86].

In a retrospective case–control study in children and adolescents with low BMD, who had no osteogenesis imperfecta and no metabolic bone disease, cumulative dose of oral alendronate was compared with DXA changes. The results indicated that alendronate does not improve bone density in children with primarily neuromuscular disease and without osteogenesis imperfecta compared to control patients with no treatment at all [87].

In a randomized placebo-controlled, multicenter trial, daily risedronate 2.5 or 5 mg for 1 year, in children 4–15 years with osteogenesis imperfecta, increased significantly lumbar spine BMD compared to control (p < 0.0001). The rate of clinical fractures decreased significantly as well after 1 year of therapy (p = 0.045). But in the follow up in years 2 and 3, the rate of clinical fractures did not change significantly in patients treated with risedronate compared to placebo-treated controls [88].

Results of these studies, and also safety issues, suggest that bisphosphonate therapy should be discontinue after 1–2 years of therapy.

### Glucocorticoid-induced osteopenia

Glucocorticoid-associated osteoporosis in growing children is associated with a 20% increase in age-adjusted fracture rates [68,69]. Glucocorticoids inhibit bone formation by decreasing the number of osteoblasts and increasing the rate of bone resorption by stimulating osteoclasts. Glucocorticoids decrease intestinal absorption and increase the renal excretion of Ca. In adults, a daily dose less than 7.5 mg glucocorticoids have been linked to such changes. In adults, alendronate effectively prevents glucocorticoid-induced osteoporosis [7,89]. Data from uncontrolled studies, in children receiving glucocorticoids treated with bisphosphonates have demonstrated significant increases in spinal bone density Z-scores after intravenous bisphosphonates or oral alendronate [90]*.* In a small placebo-controlled study in children with chronic illnesses treated with glucocorticoids, oral alendronate 1-2 mg/kg body weight/week for one year was well tolerated and increased bone density and volumetric size of the lumbar spine, and decreased indexes of bone resorption but did not improve bone growth and the size of long bones [91]. Severe corticoid-induced bone loss in Duchenne muscular dystrophy was reduced with bisphosphonate therapy [59,60].

### Pediatric bone malignancies

Pamidronate and zoledronic acid are the most rigorously studied N-bisphosphonates in adult malignancies, in addition to reducing pain and reducing fracture incidence in bone metastatic cancers, they have also an antitumor activity. In preclinical trials in pediatric cancers, Zoledronic acid was used as an active anticancer agent, with low incidence or no significant side effects in neuroblastoma [19,20], osteosarcoma [92], and in Ewing’s sarcoma [93,94].

In a phase I clinical trial in children with recurrent/refractory neuroblastoma, zoledronic acid combined with cyclophosphamide resulted in prolonged disease stability in about 50% of cases. The maximum tolerated dose of 4 mg/m2/every 28 days was recommended since it was more effective than 2 mg/m2 dose. Most side-effects were transient and tolerable. However one case of osteosclerosis complicated by fracture was reported in this study [21].

In retrospective studies [75], pediatric patients with skeletal metastatic malignancies were treated at least with one dose of intravenous zoledronic acid in combination with chemotherapeutic regiments. Patients above 10 years age received the recommended adult dose of 4 mg/kg and those younger than 10 received between 0.08 – 0.16 mg/kg. Results of these studies confirmed that zoledronic acid alleviates pain in metastatic bone cancers, has the potential antitumor actions, and has low incidence of side effects.

In Ewing sarcomas, experimental and preclinical trials have indicated the benefit of bisphosphonates as adjuvant therapies [95]. *In vitro*, zoledronic acid significantly inhibits Ewing sarcoma [94] and osteosarcoma [92] cell lines invasiveness and cell cycles [93]. In mouse models, zoledronic acid inhibits the tumor development of Ewing sarcoma in bone and reduces the dissemination of lung metastasis. However zoledronic acid neither opposes the growth of already established metastasis, nor opposes the progression of the tumor in soft tissues in Ewing sarcoma models [94].

In preclinical animal models and *in vitro* studies of neuroblastoma, zoledronic acid stimulates natural killer T cells, thus inhibiting the growth of neuroblatoma tumor cells [20], and in combination with chemotherapeutic agents, both prevents and reduces bone metastasis [19]. A phase I study for new approaches in therapy of neuroblastoma (NANT) has concluded that 4 mg/m2/28 days zoledronic acid combined with cyclophosphamide will result in stability of most cases of neuroblastomas [21].

In retrospective studies on osteonecrosis in children related to chemotherapy, 1 year pamidronate [96] or on osteonecrosis unrelated to chemotherapy, one year zoledronic acid [97], improved pain, BMD, and motor function and opposed joint destruction.

Side-effects reported in pediatric bone malignancy are those already expected. These include initial phase reaction [29,32,33], transient hypophosphatemia and hypocalcemia, prevented with supplementation with calcium and vitamin D [21,75].

Because of long half-life of zoledronic acid and secondary release of compounds, and possible inhibition of growth of long bones, long term safety is the major concern. Overall the improvement of the survival in pediatric cancer patients has yet to be determined and further study is needed. A French multicenter randomized phase III trial (OS2006- NTC00470223) is currently under way that might lead to establish appropriate pediatric dosages in bone malignancy.

### Secondary amenorrhea in young girls

*Anorexia Nervosa* is associated in nearly 50% with severe bone loss, preferentially in the spine [84]. There is a relative hypogonadism, low IGF-1, relative hypercortisolemia, low leptin, and increase adiponectin. Limited data for anorexia nervosa [34] indicates that bisphosphonate treatment reduces bone turnover and increases bone density. Risedronate 35 mg/week for 12 months administered to adult female with anorexia nervosa, has improved significantly bone densities of spine and femoral neck [84]. Oral alendronate 10 mg daily in randomized placebo-controlled trial, has also increased significantly the femoral neck and lumbar spine BMD from baseline in adolescents with anorexia nervosa vs placebo-treated patients [98]. Estrogen/progestin therapy is ineffective in preventing or reversing bone loss in girls with anorexia nervosa [99]. Recombinant hIGF-1 increases BMD but does not increase BMD to normal levels and it still an experimental strategy [100–102]. Testosterone therapy in adults female does not improve bone density [34,84] and should not be administered in young girls with anorexia nervosa. Weight gain in anorexia nervosa and exercise reduction in athletic girls typically leads to the restoration of menses [62,64].

Overall there are limited data supporting improvement of bone loss by bisphosphonate therapy in secondary amenorrhea in adolescent girls. Thus, because of unknown teratogenic effects on the fetus, bisphosphonates use in adolescent girls with secondary amenorrhea is questionable [34,62,98].

### Histiocytosis

In Langerhans cell histiocytosis, a retrospective study conducted in Japanese children aged 2.3–15.0 years, pamidronate administered intravenously at a median dose of 1.0 mg/kg/day for 3 days per cycle, between six to ten cycles at 4 to 8-week intervals, appeared to resolve the lesions in bone, skin, and soft tissues. In this study, one case of acute phase reaction, one case of uveitis, and two cases of hypocalcemia were reported in 16 children treated with pamidronate [30].

### McCune-albright syndrome, fibrous dysplasia

Fibrous dysplasia is a rare disorder arising from GNAS mutations that results in bone marrow cell proliferation and in fibro-osseous tissues in bone and bone marrow. Limited studies have demonstrated benefits of pamidronate improving pain and markers of bone turnover [103,104].

### Bone losses in impaired mobility

Cerebral palsy is characterized by impaired movements and posture, arising from abnormalities in the central motor neurons, and resulting in high prevalence of osteoporosis in children. Data from multiple controlled trials and meta-analysis studies in children with cerebral palsy treated with pamidronate or risedronate, concluded the treatment improved the BMD and reduced significantly the fracture rate after 1 year therapy [105,106], while in some trials the protective effects lasted beyond 4 years after the end of the 1-year therapy [107].

In Duchenne muscular dystrophy, other condition with impaired mobility, a retrospective analysis of patients with vertebral compression fractures treated with pamidronate or zoledronic acid demonstrated improvement in pain, and in BMD in vertebra. Prolonged glucocorticoid therapy in DMD combined with severe muscle weakness and impaired motility resulted in severe bone loss and vertebral compression fractures that was treated with bisphosphonate therapy [59,60].

Spinal cord injuries are characterized by prolonged immobilization and muscle atrophy and result in rapid bone loss and high incidence of long bone fractures. Limited case reports have shown improvement of BMD with the use of bisphosphonates in spinal cord injury in adults and children [26,108].

Bisphosphonates in in young girls with thalassemia, because of possibility of future pregnancy, should be carefully considered [71,78,109].

## Conclusion

In summary simple non-nitrogen-containing bisphosphonates result in accumulations of toxic products that lead to osteoclastic cell death while nitrogen-containing bisphosphonates inhibit osteoclast functions and activity. Both types of bisphosphonates reduce bone turnover, while the rate of bone resorption is more reduced than the rate of bone formation. The degree of avidity for the bone tissue of particular bisphosphonate determines the duration of action of the bisphosphonate after discontinuation. While long-acting bisphosphonate are a better choice for adults, they might be an undesired choice for children with growing bone. Because of possible unknown teratogenic effects, the long half-life make the bisphosphonate a questionable medication for young girls. There is not sufficient long-term efficacy and safety data for bisphosphonate therapy in pediatric age group. However short-term use appears to improve bone density and pain in conditions such as osteogenesis imperfecta, chronic corticoid therapy, and bone malignancy.

The 2014 Cochrane Database Systemic Review confirmed the positive effects of bisphosphonates concluding that bisphosphonates increase bone density in children and adolescents with osteogenesis imperfecta. There is not enough data to conclude whether bisphosphonates improve clinical status by improving growth and functional mobility in osteogenesis imperfecta. However intravenous bisphosphonates are associated with improved fracture rate in extremities and heights of vertebras.

Rational behind the use of bisphosphonates in bone malignancies is based on selective accumulation in bone tissue, potent inhibition of bone resorption by inducing osteoclasts apoptosis and increasing osteoprotegerin (OPG) production, and antitumor effects of bisphosphonates. In pediatric cancers with bone involvement, the improvement of pain, the slowdown in metastatic progression, and stabilization of the primary disease by bisphosphonates, make these medications desirable. Multicenter randomized phase III trials for pediatric bone malignancies (French OS2006- NTC00470223) might lead to establishment of appropriate dosages, optimal methods and duration of therapy, and to determination of long term safety of bisphosphonate therapy in overall pediatric patients.

One can conclude that there is a need for more future randomized studies of bisphosphonate therapy in pediatric age group to propose better evidence based recommendations.

## Abbreviations

aBMD: areal bone mineral density
NTX: Amino-terminal telopeptide of collagen cross-links
DMD: Duchenne muscular dystrophy
OPG: osteoprotegerin
LS: lumbar-sacral
